# Analog parallel processor for broadband multifunctional integrated system based on silicon photonic platform

**DOI:** 10.1038/s41377-025-01753-w

**Published:** 2025-02-07

**Authors:** Na Qian, Defu Zhou, Haowen Shu, Ming Zhang, Xingjun Wang, Daoxin Dai, Xiao Deng, Weiwen Zou

**Affiliations:** 1 State Key Laboratory of Advanced Optical Communication Systems and Networks, Intelligent Microwave Lightwave Integration Innovation Center (imLic), Department of Electronic Engineering, Shanghai Jiao Tong University, Shanghai, China; 2 State Key Laboratory of Advanced Optical Communications System and Networks, Department of Electronics, School of Electronics Engineering and Computer Science, Peking University, Beijing, China; 3 State Key Laboratory of Extreme Photonics and Instrumentation, College of Optical Science and Engineering, International Research Center for Advanced Photonics, Zhejiang University, Hangzhou, China

**Keywords:** Microwave photonics, Integrated optics

## Abstract

Sharing the hardware platform between diverse information systems to establish full cooperation among different functionalities has attracted substantial attention. However, broadband multifunctional integrated systems with large operating frequency ranges are challenging due to the bandwidth and computing speed restrictions of electronic circuitry. Here, we report an analog parallel processor (APP) based on the silicon photonic platform that directly discretizes and parallelizes the broadband signal in the analog domain. The APP first discretizes the signal with the optical frequency comb and then adopts optical dynamic phase interference to reassign the analog signal into 2^N^ parallel sequences. Via photonic analog parallelism, data rate and data volume in each sequence are simultaneously compressed, which mitigates the requirement on each parallel computing core. Moreover, the fusion of the outputs from each computing core is equivalent to directly processing broadband signals. In the proof-of-concept experiment, two-channel analog parallel processing of broadband radar signals and high-speed communication signals is implemented on the single photonic integrated circuit. The bandwidth of broadband radar signal is 6 GHz and the range resolution of 2.69 cm is achieved. The wireless communication rate of 8 Gbit/s is also validated. Breaking the bandwidth and speed limitations of the single-computing core along with further exploring the multichannel potential of this architecture, we anticipate that the proposed APP will accelerate the development of powerful opto-electronic processors as critical support for applications such as satellite networks and intelligent driving.

## Abstract (web summary)

The principle and architecture of analog parallel processor for broadband multifunctional integrated system.

## Graphical abstract

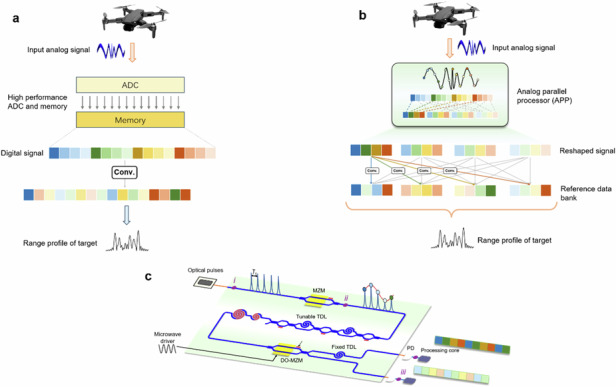

## Introduction

The effective acquisition and utilization of massive, diversified, and multifunctional information is crucial for intelligent sensing systems, such as unmanned vehicles and the Internet of Things^1–3^. To achieve this capability, the type and number of radio-frequency (RF) sensors are continuously expanding, resulting in quality and volume that exceed the affordable limit of mobile or distributed sensing platforms. Multifunctional integrated systems, which share the hardware platform among different functionalities, break the constraints of traditional resource division technologies and share resources efficiently^4^. Benefitting from technologies such as digital array antenna^5^, waveform multiplexing^6^, and resource scheduling^7^, multifunctional integrated systems achieve a higher degree of fusion and collaborative efficiency. All these strengths are in compliance with the development trends of intelligent sensing systems, especially airborne, vehicle, and other mobile platforms^8^. Future generations of multifunctional integrated systems will propel broader bandwidth to achieve increased data rate and finer sensing resolution, as well as require wider range of operating frequencies for more flexible scheduling of frequencies and functions^9–13^. However, the bandwidth and computing speed restrictions of electronic circuitry have hindered the evolution of multifunctional integrated systems towards efficient information acquisition and interaction across a wider spectrum^14^.

Microwave photonics (MWP) can access a broader bandwidth compared with electrical technologies, harnessing the processing bandwidth obtained from upconverting radio-frequencies (RF) to optical frequencies^15,16^. Broadband solutions include MWP receivers based on de-chirping for broadband linear frequency-modulated (LFM) radar echo signals^17^, integrated MWP filters for large-capacity wireless communication systems^18,19^, and MWP receivers capable of microwave signals measurement^20,21^. These solutions pursue broad acquisition bandwidth and flexible RF response tailoring capability. However, as they are designed and implemented to optimally perform a particular functionality, there is a lack of universality and reconfigurability for multifunctional applications^16^. Meanwhile, fully digital solutions that use analog-to-digital converters (ADC) along with digital signal processors (DSP) to implement multifunctional receiving and processing can guarantee universality and reconfigurability. Nevertheless, to process signals with broad bandwidth, the fully digital solution will produce a great deal of data, occupying a significant amount of memory and processing resources. This puts undue stress on high-performance signal processors^14^. Although emerging photonic signal processors excel in terms of clock rate, throughput, and power efficiency^22–30^. Their wide deployment is impeded by two challenges: access to high-speed memory to prevent a memory bottleneck^31,32^, and the total insertion loss when scaling up network dimensions^26^. In this case, how to advance broadband photonic signal processing capacity with mature electrical circuits without putting additional pressure on memories is an urgent problem to be solved.

Here, we demonstrate a silicon photonic platform-based analog parallel processor (APP) for broadband multifunctional integrated systems, which directly discretizes and parallelizes the broadband signal in the analog domain. Thus, computing cores with reduced clock rate and data volume are capable of processing diverse broadband signals. High-speed memories are also significantly omitted, which are usually configured to complete serial-parallel conversion in traditional electrical digital processors. The APP utilizes the optical frequency comb to discretize broadband analog signal, and then reassign temporal discrete signal into 2^*N*^ parallel sequences through optical dynamic phase interference. For each sequence, the data is demultiplexed in time-domain and processed independently through low-speed computing cores in each channel. It is verified that the fusion of outputs from each computing core is equivalent to processing broadband signals directly. In the proof-of-concept experiment, a two-channel APP chip is fabricated. The universality of APP for multifunctional applications is validated through processing broadband radar signals and high-speed communication signals on the single photonic integrated circuit. Its high-resolution-ranging capability is experimentally demonstrated with a resolution up to 2.69 cm. The accurate detection of communication signals with complex modulation format guarantees a communication rate of 8 Gbit/s.

**Fig. 1:**
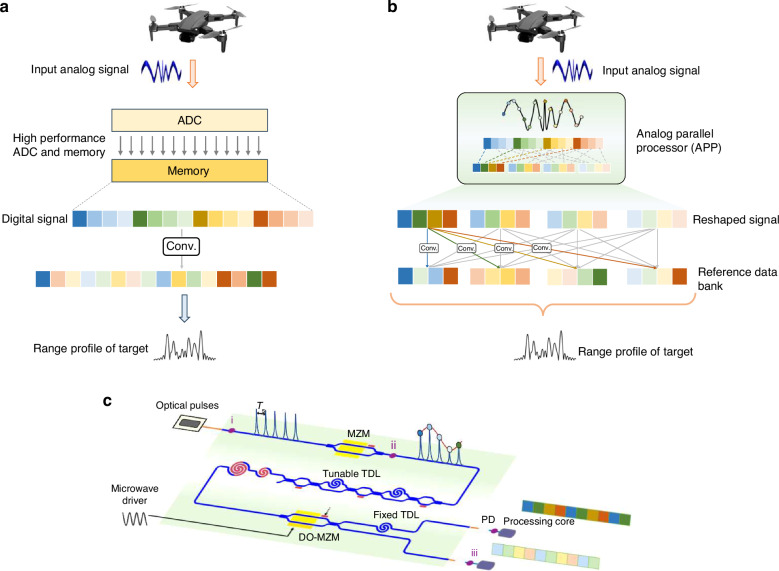
The principle and architecture of APP. **a** The workflow of traditional digital processing for multifunctional integrated systems, including high-speed ADCs, large-capacity memories, and high-performance computing cores. **b** The workflow of APP. The input analog signals are temporally discretized and routed into 2 ^N^ parallel sub-sequences in the APP chip. The data rate and volume in each sub-sequence are reduced exponentially. The coefficients of multipliers are decomposed in the same order into 2^N^ channels. Multiplication of signal and coefficients in each channel is performed respectively. The output results from each channel are added in pairs to achieve the complete processing result. **c** Schematic of the APP chip. MZM mach-zehnder modulator, TDL time delay line, DO-MZM dual-output modulator, PD photodetector. The optical pulses and MZM perform temporal discretization in the optical domain. The DO-MZM is driven by a microwave driving signal to parallelize the temporal discrete signal. Tunable TDL allows adjacent optical pulses to enter corresponding sub-channels without residual components

The signal processing for multifunctional integrated systems usually includes down-conversion, filtering, pulse compression, spectrum analysis, signal accumulation, etc^33,34^. We can decompose such operations into linear multiplication and summation with a specific order, and then implement decomposed processing onto the temporal signal sequence. As digital processing remains the mainstream, multifunctional integrated tasks usually convert the input analog signals into digital signals by ADCs. And store digital signals in memories for subsequent digital signal processing (Fig. 1a). However, for broadband multifunctional integrated systems, high-speed ADCs, and large-capacity memories are essential according to Shannon’s theorem^32^. Moreover, as the amount of data increases, the requirement of multiplications and summations also grows, thus demanding more computing resources. In the process of the APP (shown in Fig. 1b), the input analog signals are temporally discretized by the optical frequency comb. The optical frequency comb with a high repetition rate realizes high-speed optical discretization. Optical discretization is the pre-processing for parallelization. After the optical discretization, the signals are parallelized through the periodic optical switching window. Temporal discrete signals are routed into 2^*N*^ parallel sub-sequences, thereby reducing the effective data rate and volume in each computing core. To match the parallel sub-sequences, the coefficients of multipliers are decomposed in the same order and spatially divided into 2 ^*N*^ channels. Multiplication of signal and coefficients in each channel is performed respectively, with the output results from each channel added in pairs to achieve the complete processing result. The clock rate and data volume of each computing core are therefore 2^*N*^-time reduced. Memory access rate is also significantly compressed, with the elimination of serial-parallel conversion in traditional electrical parallel processors. Here we take pulse compression in radar signal processing as an example, which is calculated by the convolution of echo analog signal *s*(*t*) and the conjugate function of transmitting signal *h*(*t*). The computation of pulse compression is represented by:

1 $$y(n)=h(n)\ast s(n)$$

where *h*(*n*) denotes the conjugate function of transmitting signal, *s*(*n*) denotes the echo analog signal, and *y*(*n*) denotes pulse compression output. The equivalent computation in APP is expressed as:

2 $$\begin{array}{c}{y}^{i,j}={h}_{i}\ast {s}_{j}\\ y(n)=\mathop{\sum }\limits_{k=0}^{K-1}{y}^{k,(i-k)\mathrm{mod}K}\end{array}$$

where *h*_*i*_ and *s*_*j*_ represent the *i*th sub-sequence of *h*(*n*) and the *j*th sub-sequence of *s*(*n*), respectively. *y*(*n*) is the final fused pulse compression output. *K* stands for the number of *h*_*i*_ or *s*_*j*_. *s*_*j*_ is generated through the APP chip in the optical domain without memories and controllers for serial-parallel conversion. Detailed descriptions of other calculation decomposing procedure can be found in Supplementary Note 1.

Following the APP concept, the schematic of the photonic integrated chip is illustrated in Fig. 1c. The optical pulses at the repetition interval of *T*_*S*_ are generated by a mode-locked Er-fiber optical frequency comb and coupled to the chip by an on-chip edge coupler. The analog signal is modulated on the optical pulses through the Mach-Zehnder modulator (MZM) biased at its quadrature point, which is temporally discretized for parallelization in the optical domain. Here, the dual-output Mach-Zehnder modulators (DO-MZM) is employed to parallelize the temporal discrete signal^35^. Two outputs of the DO-MZM correspond to two sub-sequences after each stage parallelization. The principle of optical dynamic phase interference in Supplementary Note 2 reveals a fixed phase difference π between envelopes of optical pulses from each sub-sequence, indicating analog parallelizing based on DO-MZM is inherently phase uniform. Uniform parallelizing without RF phase offset between sub-sequences is critical for the accuracy of analog processing^36^, and is difficult to be accomplished in electrical domain due to the wiring error, especially when bandwidth increases. Specifically, the tunable optical delay line (TDL) is added before the DO-MZM to allow two adjacent optical pulses to pass through the DO-MZM at the maximal and the minimal intensity transmission levels, respectively^37^. The DO-MZM is driven by a driving signal at the frequency of *f*_*d*_ (*f*_*d*_ = 1/2*T*_*S*_) to achieve optical dynamic phase interference. Subsequently, two sub-sequences are aligned via a fixed delay line for synchronization. Photodetectors (PDs) convert the signal in each sub-sequence to electrical signals. Low-speed ADCs and DSPs in each channel perform decomposed computation, subsequently, the complete processing result is achieved through fusing outputs from each channel. The schematic output sequence of each step on our chip is illustrated in Fig. 1c.

## Results

### The fabricated analog parallel processor chip

**Fig. 2:**
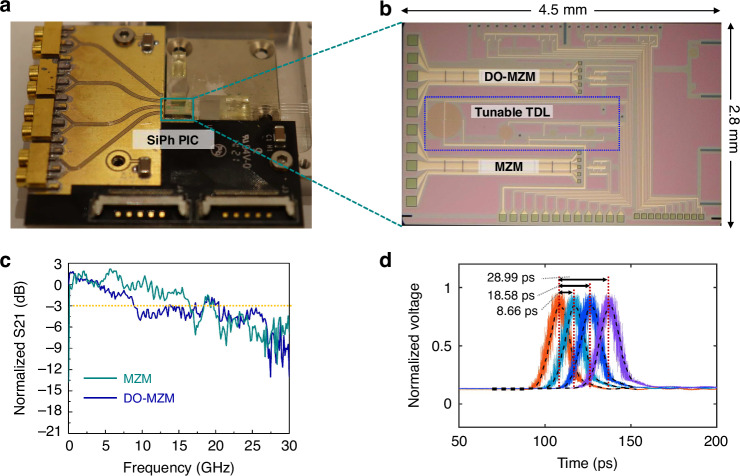
Chip fabrication and characterization. **a** Photograph of the packaged APP chip. Optical signals enter and leave the chip via the edge-coupled fiber. **b** Layout of the APP chip. The analog signal is temporally discretized through the MZM. The temporal discrete signal is parallelized into two parallel sub-channels by the DO-MZM, with the aid of tunable TDL. **c** Normalized measured EO modulation responses of the packaged MZM and DO-MZM. **d** Measured optical output waveforms with a time delay of 8.66 ps (light blue curve), 18.58 ps (dark blue curve), and 28.99 ps (purple curve). Here the orange curve is the measured reference optical output waveform passing through the corresponding straight waveguide

Figure 2a shows the photograph of the APP chip, which is fabricated with a standard Silicon-on-Insulator (SOI) integration process. We implement most of the required optical components onto the chip, including modulator, tunable TDL, fixed TDL, and DO-MZM. The fabricated chip conducts two-channel parallel processing with a discrete data rate of 40 GHz. Duplicating the same structure can expand the number of sub-channels and thus increase the total data rate and data volume. Optical signals enter and leave the chip through the waveguide-fiber edge coupler. Figure 2b depicts the layout of the fabricated APP chip, comprising a modulator, a tunable TDL and a DO-MZM. Figure 2c presents the measured electro-optic (EO) modulation responses of the packaged MZM and the DO-MZM. The EO bandwidth of the MZM ultimately determines the analog input bandwidth of the whole processor. The bandwidth of the DO-MZM determines the frequency range of the driving signal for optical dynamic phase interference and further affects the total data rate^38^.

As shown in Fig. 2c, the measured 3 dB EO bandwidth is 16.5 GHz and 9.1 GHz for MZM and DO-MZM, respectively. This difference is the result of different reverse-bias voltages applied to PN junctions, which are independently adjusted to match the amplitude of analog input signal onto the MZM and driving signal onto the DO-MZM^35^. Figure 2d illustrates the characterization result of tunable TDL showing the measured time delay tuning range of 28.99 ps. The discrete data rate of this APP chip is 40 GHz and the repetition interval of optical discrete points is 25 ps, Thus, the on-chip delay tuning range is sufficient. Here, the orange curves are the reference optical output waveforms passing through the corresponding straight waveguide. The light blue, dark blue and purple curves in Fig. 2d are optical output waveforms passing through one waveguide spiral with a designed time delay of 10 ps, one waveguide spiral with a designed time delay of 20 ps, and two waveguide spirals with a total designed time delay of 30 ps, respectively. The difference between the measured value and the designed value is mainly due to the fabrication error. Detailed information on photonic device design and characterization can be found in Supplementary notes 3–4.

### Optical discretization and parallelization

**Fig. 3:**
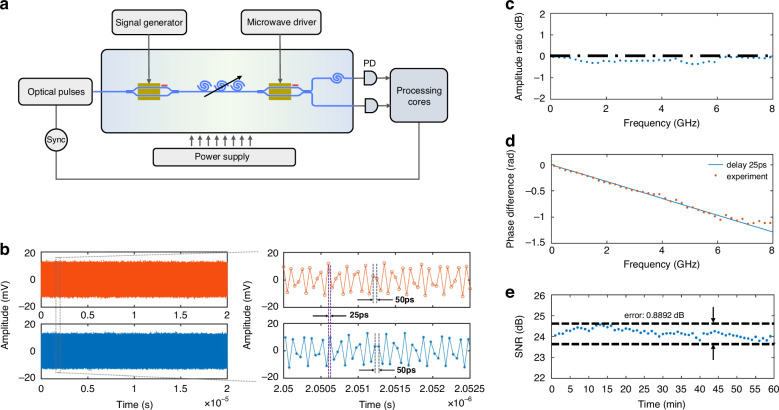
Experimental results of optical discretization and parallelization. **a** Experimental setup to measure the optical discretization and parallelization capability of the APP chip. PD photodetector, Sync synchronization. **b** Temporal outputs from two sub-channels when the input single-tone signal is 8.1 GHz. **c** Measured amplitude ratio response of two sub-channels. **d** Measured phase difference frequency response between two sub-channels. **e** Measurement of the SNR over 1 h continuous operation

Figure 3a illustrates the experimental setup for validating the optical discretization and parallelization capability of the APP chip (see the “Materials and methods” section for details of the experimental setup). The single-tone signal provided by an analog signal generator is launched to the chip. Figure 3b shows the temporal outputs from two sub-channels, where the frequency of the input single-tone signal is 8.1 GHz. The orange and blue curves represent sub-channel 1 and 2, respectively. After discretization and parallelization, the analog input signal is uniformly converted into two parallel discrete sequences. From the zoom-in plot, the delay between adjacent data points is 50 ps in each sub-channel, corresponding to the data rate of 20 GHz. The delay difference between the two sub-channels is depicted as 25 ps. It means the original discretized signal is temporally divided and reshaped into 2 channels.

Since the discrete points in each sub-channel are processed independently and then fused to achieve the results, it is necessary to ensure the amplitude and phase uniformity between the two sub-channels. We measure the amplitude ratio and phase difference of two sub-channels under different input frequencies by gradually tuning the frequency of analog input signal. The output amplitude ratio response of two sub-channels is depicted in Fig. 3c. The measured amplitude ratio is under 0.36 dB within the measured frequency band, which demonstrates great consistency. Taking sub-channel 1 as a reference, the phase difference frequency response is measured and displayed in Fig. 3d. The experimental results (orange dot) are in good agreement with the theoretical phase difference frequency curve for a time delay of 25 ps (blue line). The phase-frequency curve is slightly fluctuant due to some practical limitations such as the nonlinear phase response of off-chip PDs^39^. The measured amplitude ratio response and phase difference response indicate that these two sub-channels match well in both amplitude and phase. This benefits efficient parallel processing and avoids complex inter-channel calibrations, which are more cumbersome for broadband signals.

The long-term stability of our chip is also evaluated by monitoring the real-time signal-to-noise ratio (SNR) over a 1-hour experiment. The SNR is calculated by the ratio of signal power to integrated noise power in full Nyquist bandwidth. As presented in Fig. 3e, the calculated SNR shows acceptable fluctuations (<0.9 dB), which reveals that photonic integration of main optical components leads to great advances on the robustness of environmental perturbations^20^.

### Broadband multifunctional integrated demonstration using the APP chip

**Fig. 4:**
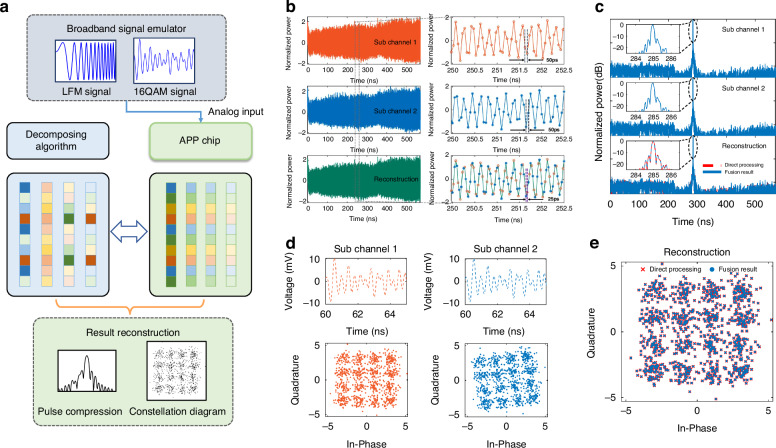
Experimental results of broadband multifunctional integrated demonstration using the APP chip. **a** Structure of the broadband signal parallel processing. The input analog signals are temporally discretized and parallelized in the APP chip. The algorithm and coefficients of multipliers are decomposed in the same order. Output results from each channel are combined in pairs to reconstruct the complete processing result. **b** The temporal waveforms from each sub-channel and reconstruction result. The data rate of each sub-channel is 20 GHz and the data rate of reconstructed waveform is 40 GHz. **c** Pulse compression results of each sub-channel; fusion result of two sub-channels (blue solid line in the bottom); direct processing result (red dashed line in bottom). **d** Temporal waveforms and constellation diagrams of sub-channel 1 (orange lines) and sub-channel 2 (blue lines). **e** Fused constellation diagram of two sub-channels (blue dots); direct processing result (red cross dots)

Based on the successful validation of optical discretization and parallelization, we move on to process multiple-waveform broadband signals for different functions, such as radar and communication^40,41^. To validate the broadband signal processing capacity of our APP chip, we apply it to process broadband LFM radar signals and communication signals in complex modulation formats. Figure 4a displays the structure of the broadband signal parallel processing and the experimental setup is similar to Fig. 3a (details are provided in “Materials and methods”). The input analog signals are temporally discretized and parallelized into sub-sequences with lower data rates in the APP chip. The algorithm and coefficients of multipliers are decomposed in the same order. Output results from each channel are combined in pairs to reconstruct the complete processing result. The instantaneous bandwidth of LFM signals is the determining factor for range resolution in radar function. We generate LFM signals with 6 GHz bandwidth as simulated radar echo signals. The temporal waveforms of LFM signals from each sub-channel and reconstruction result are shown in Fig. 4b. It is observed from the zoom-in plot that the delay between adjacent data points is 50 ps, in each sub-channel. Moreover, the reconstructed waveform is reassembled by rearranging the data from two sub-channels one by one. The range resolution is usually used to characterize the radar detection ability, which is calculated by pulse compression. Here, we conduct the pulse compression by calculating the autocorrelations of obtained temporal waveforms. The pulse compression results are depicted in Fig. 4c. As shown in the zoom-in insets, the experimental resolution is 2.69 cm, which agrees well with theoretical values (2.5 cm for 6 GHz bandwidth LFM signal). The result reveals that the fusion of pulse compression outputs from each sub-channel (blue solid line in Fig. 4c bottom) is consistent with directly processing broadband LFM signal (red dashed line in Fig. 4c bottom). The APP chip owns a flexible processing capability in the range of 2–8 GHz (covering the whole S-band and C-band). Also, we demonstrate the ability to detect multiple targets at multiple distances, Supplementary note S7 describes the details and results of this demonstration. It verifies the feasibility of our APP chip for acquiring high-resolution range profiles. Moreover, it is expected to serve as a processor in inverse synthetic aperture radars for precise target imaging, by employing the coherent accumulation of radar echo signals^42^.

The APP chip further shows flexible processing capability in terms of complex communication signals. The 16-quadrature amplitude modulation (16QAM) signal based on orthogonal frequency division multiplexing (OFDM) is fed into the chip. The bandwidth of the 16QAM signal is 2 GHz, for which the frequency spacing of adjacent subcarriers is 1 MHz and the number of orthogonal subcarriers is 2000. The net communication rate of the 16QAM signal is 8 Gbit/s. The temporal waveforms of sub-channel 1 (orange lines) and sub-channel 2 (blue lines) are recorded in Fig. 4d. Based on temporal waveforms from the two sub-channels, the constellation diagrams after demodulation for each sub-channel are plotted in Fig. 4d. The calculated BER is 1.375 × 10^-2 for sub-channel 1 and 1.35 × 10^-2 for sub-channel 2, no extra error correction adopted. We then combine the demodulation results from sub-channels and plot the fused constellation diagram in Fig. 4e (blue dots). The direct processing result is also plotted in Fig. 4e (red cross dots). The comparison in Fig. 4e shows excellent equivalence of our chip in analog parallel processing. The calculated BER of the combined result is 1.375 × 10^-2. Overall, for advanced communication scenarios, our chip exhibits its broadband and high-speed processing capability.

## Discussion

In multifunctional integrated systems, universal processors with ultrabroad bandwidth and high computational accuracy are the common pursuit. We note that the proposed APP chip has the potential to be upgraded toward an ultrabroad one. Recent breakthroughs in electro-optic integrated modulators, on conventional SOI platforms or thin-film lithium niobate platform^43–49^, pave the way for a broad modulation bandwidth. Replacing the modulators used in the proposed chip can greatly enhance the analog input bandwidth. On the other hand, to increase the total data rate, cascaded DO-MZMs for constituting more sub-channels are inevitable. In Supplementary material, potential scalability, and total data rate enhancement of APP chip are discussed. As the number of sub-channels goes to 2 ^*N*^, the total number of tunable TDLs and DO-MZMs both increases to 2^*N*^-1, and the insertion loss becomes the primary limitation of chip implementation with the current silicon photonic platform. More critically, the accumulated insertion loss leads to the deterioration of SNR and computational accuracy, which adversely affects the sensitivity in radar systems and the order of modulation format in communication systems. Therefore, in future engineering, the insertion loss of the APP chip should be refined significantly from the following aspects: using advanced fiber-chip coupling^50^; using low-loss components; using loss compensated modulators based on heterogeneous platform^51^; using on-chip amplifier^52^; using on-chip PD to avoid chip-fiber coupling. The whole parallel processing system is supposed to be integrated on the chip towards real-world application scenes, including the optical pulses, optical amplifiers, PDs, and processing cores. With the recent development of heterogeneous or hybrid integration technologies, combining strengths from diverse material platforms and being compatible with CMOS electronics have proven to be a viable paradigm^20,53–60^. Moreover, cavity-less ultra-short optical pulses based on cascaded modulators, which can produce high-speed optical pulse trains, would also be used to generate optical pulses for temporal discretization. Integrated modulators based on thin-film lithium niobate platforms are beneficial for reducing the size and power consumption of cavity-less optical pulses. It is foreseeable that the SWaP and robustness of the APP chip will be further optimized.

The range resolution is usually used to characterize the radar detection ability, which depends on the bandwidth of LFM radar signals. The relation is Δ*r* = *c*/2*B*, where ∆*r* denotes the range resolution and *B* is the signal bandwidth. To further improve the range resolution, the analog input bandwidth of the APP chip can be upgraded by expanding the electro-optic bandwidth of the MZM. Besides, our APP chip is capable of processing analog echo signals directly, transforming the analog input signals into parallel sequences with reduced data rate and data volume. These characteristics allow for the processing of larger bandwidth signals to achieve finer resolution, without putting stress on high-speed ADCs and large-capacity memories. Moreover, the signal processing for multifunctional integrated systems can be divided into the combination of dot product, convolution, and basic vector operation as derived in Supplementary notes 1. Thus, although our proof-of-concept experiment was validated for radar and communication signals, the APP chip would be effective for more kinds of signals and improve the collaborative efficiency in multifunctional integrated systems. The direction of arrival estimation, which is a critical technology for multifunctional integrated systems, also needs to develop more effective computing paradigms beyond conventional array signal processing methods^61,62^.

In conclusion, we propose an integrated analog parallel processor for multifunctional integrated systems. Compared with mainstream digital processors, it is capable of processing broadband signals without extra digital data parallelization and memory use. The optical frequency comb temporally discretizes broadband analog signal and the DO-MZM reshapes discrete signal into 2 ^*N*^ parallel sequences through optical dynamic phase interference. After optical discretization and parallelization, the signal in each sequence is processed by computing cores with reduced clock rate and data volume. The limitation of memory in traditional digital serial-parallel conversion is thus eliminated. In the proof-of-concept experiment, an APP chip based on the silicon photonic platform is fabricated. It performs two-channel parallel processing, exhibiting good amplitude and phase uniformity between two sub-channels. The multifunctional integrated processing is carried out with a ranging resolution of 2.69 cm and a communication rate of 8 Gbit/s. Since the cooperation and mutual promotion of multiple functions have become the main trend, our APP chip will become an effective solution for advanced information systems such as electronic warfare, autonomous driving, and the Internet of Things.

## Materials and methods

### Experimental setup

The schematic of the experimental setup for optical discretization and parallelization is illustrated in Fig. S5. In the experiment, the optical pulses are generated by the mode-locked Er-fiber optical frequency comb (Calmar PSL-40-TT) with a repetition rate of 40 GHz. The single-tone signal provided by an analog signal generator (Rohde&Schwarz SMA100B) is launched to the on-chip MZM with the aid of an RF balun (Marki BAL-0026). The microwave driving signal (Keysight Technologies, N5183B) for driving the DO-MZM is at 20 GHz and strictly synchronized with the mode-locked Er-fiber optical frequency comb. An RF balun (Marki BAL-0026) is also used to differentially drive the two EO phase shifters of the DO-MZM. Both the MZM and the DO-MZM are biased at the quadrature point. Their desired bias voltages are provided by a home-made multi-channel voltage source and combined with RF signals using bias-tees (Marki BT-0025). For on-chip tunable TDL, the thermal controlling of MZ switches and spiral lines is also supplied by the home-made multi-channel voltage source. The parallel pulses in each sub-channel are amplified by erbium-doped optical fiber amplifiers (EDFA, ASPMEDFA-C-BA-20) and subsequently detected into electrical sequences by PDs (EOT ET-3500F). The gain of the EDFA is 30 dB. The bandwidth and responsivity of PDs is 10 GHz and 0.9 A/W @1550 nm, respectively. A real-time oscilloscope (Keysight Technologies, MSOS804A) is used as EADCs to quantize two electrical sequences, where the oscilloscope has a quantizing rate of 20 GSa/s and an input analog bandwidth of 8.4 GHz.

The process for conceptual multifunctional integrated processing is shown in Fig. 4a, and the experimental setup is similar to that in Fig. S5. In the broadband signal emulator, an arbitrary waveform generator (AWG, Keysight Technologies, M8195A, 65GSa/s) is employed to generate broadband LFM radar signals and complex modulated communication signals. The generated signals are first amplified by a power amplifier (CONNPHY CLN-0.1G20G-3040-S), and then fed into the on-chip MZM. After optical discretization and parallelization, parallel pulses in both sub-channels are amplified by EDFAs (ASPMEDFA-C-BA-20) and converted into electrical sequences by PDs (EOT ET-3500F). Afterwards, the electrical sequences are quantized by the real-time OSC (Keysight Technologies, MSOS804A). The pulse compression and demodulation for each sub-channel as well as the reconstruction for fusion results are implemented by our computer.

### Package

The packaged APP chip is shown in Fig. 2a. For optical packaging, customized tapered fiber is employed to match the input fiber waveguide and silicon waveguide mode field, and they are fixed by using ultraviolet curing adhesive. For electrical connections, the RF and DC pads on the chip are wire-bonded to a custom-designed print circuit board (PCB). As the wire bonding for RF connections is critical to the behavior of our chip at high frequencies, we maintain the length of gold wire as short as possible and suppress the crosstalk between adjacent RF channels.

### Insertion loss of the APP chip

The insertion loss of the APP chip is mainly composed of several parts: fiber-chip edge coupling, MZM, tunable TDL, DO-MZM, and the waveguide loss from the input to the output of the chip. The insertion loss of fiber-chip edge coupling is around 6.5 dB/facet. It is ~8 dB for the MZM and DO-MZM operating at the quadrature bias point. In the proof-of-concept experiment, the optical power injected into the APP chip is about 13 dBm, and the average output power from each sub-channel is about -22 dBm. In summary, the measured insertion loss of the current version of the APP chip is ~35 dB for each sub-channel, including the coupling loss.

### Time delay measurement of tunable TDL

To experimentally characterize the tunable TDL, a pulse generated by a mode-locked Er-fiber optical frequency comb (Calmar PSL-40-TT) at 1550 nm is launched to the tunable TDL and received by a wide-bandwidth oscilloscope (Keysight Technologies, Infiniium DCA-X 86100D). The time interval resolution of this oscilloscope is ≤ 62.5 fs^63^. The MZ switches in the tunable TDL are switched thermally by heating micro-heaters controlled by a home-made multi-channel voltage source^64^. Here, we measure optical output waveforms passing through the corresponding straight waveguide as reference (orange curve in Fig. 2d). Optical output waveforms, passing through one waveguide spiral with the designed time delay of 10 ps, one waveguide spiral with the designed time delay of 20 ps and two waveguide spirals with total designed time delay of 30 ps, are also measured and recorded (represented by light blue, dark blue, and purple curves in Fig. 2d). The time delay can be calculated through recorded waveforms.

### Amplitude ratio and phase difference measurement of two sub-channels

We quantitatively evaluate the amplitude and phase uniformity between two sub-channels. Optical discretization and parallelization on the APP chip have been regulated to the proper state^35^. The gain of two off-chip EDFAs and the length of tail fibers are consistent. We input the single-tone signal to the APP chip. The frequency range of the test single-tone signal is 0.1 GHz-7.9 GHz, with a step of 200 MHz. At each frequency, the electrical sequences after quantization in each sub-channel are collected. Fourier transform is then performed and digital spectra for both channels are obtained. We extract amplitude and phase from the digital spectra at each frequency. The amplitude ratio and phase difference of two sub-channels under different input frequencies are calculated and plotted in Fig. 3c and d.

### Parallel processing of LFM and 16QAM signal

LFM signals are used in most radar systems to achieve wide operating bandwidth. As a method for improving the range resolution of pulse radar, pulse compression performs a correlation between the received echo signal and the transmitting reference signal, which looks for a strong correlation between them. Traditional pulse compression is calculated by the convolution of the echo signal and the conjugate function of the transmitting signal. With our APP chip, the pulse compression is accomplished by the following steps: (1) obtaining two parallel discrete sequences from our chip; (2) performing the Hilbert transform on the data of two sub-channels independently, and removing the carrier frequency to get *s*_1_ and *s*_2_; (3) generating the reference signal according to the pulse duration and operating frequency range of echo signal; (4) conjugating the reference signal and separating it into two sequences *r*_1_ and *r*_2_; (5) obtaining four sets of convolution results between two echo sequences and two reference sequences: *Cr*_1_*s*_1_, *Cr*_2_*s*_2_, *Cr*_1_*s*_2_, and *Cr*_2_*s*_1_. To achieve the fused pulse compression, we sum up four sets of convolution results according to Eq.2.

QAM signal transmits information by changing both the amplitude and phase of carrier waves, thereby doubling the effective bandwidth. It is thus essential to demodulate 16QAM signals through Fourier transform to obtain symbol information. With our APP chip, the original Fourier transform is deformed and disassembled into two smaller Fourier transforms. Perform Fourier transform on two sub-sequences respectively. According to the basic properties of the Fourier series, the Fourier transform of original 16QAM signals is equivalent to the sum of the Fourier transforms of two sub-sequences, where the Fourier transform of one sub-sequence is multiplied by the phase rotation factor^65^. Map the demodulated symbol information (including amplitude and phase information) to polar coordinates. Constellation diagrams represent two-dimensional QAM modulation information, the distance from the point to the origin (0,0) representing the amplitude after modulation, and the angle of the point representing the phase after modulation.

## Supplementary information

Supplementary Information for Analog parallel processor for broadband multifunctional integrated system based on silicon photonic platform

## Data Availability

All data used in this study are available from the corresponding authors upon reasonable request.
