# Supplementary material for: Analog parallel processor for broadband multifunctional integrated system based on silicon photonic platform
Source: Light Sci Appl. 2025 Feb 7;14:71. doi: 10.1038/s41377-025-01753-w (PMC11802829; doi:10.1038/s41377-025-01753-w)
Supplement: Supplementary file 1 — Supplementary Information for Analog parallel processor for broadband multifunctional integrated system based on silicon photonic platform [file 41377_2025_1753_MOESM1_ESM.docx]

**Supplementary Information for**

**Analog parallel processor for broadband multifunctional integrated system based on silicon photonic platform**

Na Qian^1^†, Defu Zhou^1^†, Haowen Shu^2^, Ming Zhang^3^, Xingjun Wang^2^, Daoxin Dai^3^, Xiao Deng^1^, Weiwen Zou^1*^

*^1^State Key Laboratory of Advanced Optical Communication Systems and Networks, Intelligent Microwave Lightwave Integration Innovation Center (imLic), Department of Electronic Engineering, Shanghai Jiao Tong University, Shanghai 200240, China*

*^2^State Key Laboratory of Advanced Optical Communications System and Networks, Department of Electronics, School of Electronics Engineering and Computer Science, Peking University, Beijing 100871, China*

*^3^State Key Laboratory of Extreme Photonics and Instrumentation, College of Optical Science and Engineering, International Research Center for Advanced Photonics, Zhejiang University, Hangzhou 310058, China*

**Correspondence to:* [*wzou@sjtu.edu.cn*](mailto:wzou@sjtu.edu.cn)

†*These authors contributed equally to this work*

Supplementary notes S1-S7;

Supplementary figures S1-S6;

Supplementary table S1;

Supplementary references S1-S8.

**Supplementary notes**

**S 1 Calculation** **decomposing for analog parallel processing**

The proposed APP discretizes and parallelizes the broadband signal in analog domain directly, thus reduces the data rate and data volume for each processing core in multifunctional integrated systems. The functions of a multifunctional integrated system usually include radar, communication, positioning, spectrum measurement, analysis, etc. The signal processing of these functions can be disassembled as down-conversion, filtering, pulse compression, Fourier transform, etc. These processing processes can be divided into the combination of dot product, convolution, and basic vector operation. Therefore, we decompose such operations into linear multiplication and summation with the order same to the sub-sequences output from the APP chip. The calculation of dot product is given by:

$$y=h\left(n\right)\cdot s\left(n\right)$$

where *h*(*n*) denotes the coefficient vector, *s*(*n*) denotes the signal vector, *y* denotes dot product output. The decomposed calculation of dot product in APP is expressed as:

$$y=\sum_{k=0}^{K-1} h_{k}\cdot s_{k}$$

where *h_k_* and *s_k_* represent the *k*-th sub-sequence of *h*(*n*) and *s*(*n*), respectively. *y* is the final summed dot product output. *K* is the number of sub-sequences. *s_k_* is generated through the APP chip. Fig. S1(a) shows the decomposing schematic of the dot product. It is obvious that the decomposed calculation of dot product can be executed through single instruction for multiple sub-sequences simultaneously. For basic vector operation shown in Fig. S1(b), its calculation can be expressed as:

$$y\left(n\right)=k\cdot s\left(n\right)+b\cdot l\left(n\right)$$

where *s*(*n*) and *l*(*n*) denote the signal vector and constant vector, respectively. *k* and *b* are constant scalars. When *s*(*n*) is parallelized through the APP chip into sub-sequence *s_k_*, the decomposed basic vector operation is expressed as follows:

$$y_{k}=k\cdot s_{k}+b\cdot l_{k}$$

where *l_k_* is the *k*-th sub-sequence of *l*(*n*). *y_k_* is the *k*-th basic vector operation output. Therefore, the final basic vector operation output is achieved through reshaping *K* vectors *y_k_* into final vector *y*(*n*).

**S 2 Principle of optical dynamic phase interference**

The dual-output modulator (DO-MZM) is employed to parallelize the temporal discrete signal. Two outputs of the DO-MZM correspond to two sub-sequences after each stage parallelization. Outputs of the DO-MZM are expressed below:

where *I_in_* is the optical intensity into the DO-MZM, *l_0_* is the insert loss, and *φ* is the optical field phase shift of the two modulation arms. Based on optical interference, output optical intensity *I_out_*_,1_ and *I_out_*_,2_ vary between 0 and *l_0_I_in_* through manipulating the optical field phase shift *φ*. When the optical field phase shift *φ* periodically changes from 0 to 2π, there is an optical dynamic phase interference in the DO-MZM. The equation reveals a fixed phase difference π between two sub-sequences, indicating analog parallelizing based on DO-MZM is inherently phase uniform.

**S 3 Characterization of the silicon EO modulator**

The bandwidths of the cascaded modulators were characterized separately by standard VNA measurement, as depicted in Fig. S2a. The light coupled from the grating connected to the output 2x2 MMI structure of the first modulator was sent into the high-speed photodetector (PD) of test system. The PD holds a flat OE response below the frequency range of 60 GHz, thus the degradation due of its inherent bandwidth could be negligible. For the second modulator, to get avoid of prepositive link loss, all switches before the modulator, including the Mach-Zehnder interferometer based optical delay structures and the first modulator, are set to work under the perfect constructive interference point, via the tuning of on-chip phase shifters. Both devices present 3 dB bandwidth of over 25 GHz, which supports the low-loss RF operation.

**S 4 Parameter design of the tunable delay line**

A silicon tunable delay line (TDL), composed of cascading optical switches and waveguide delay lines was introduced to achieve ultra-high tuning precision of delay line [1]. These optical switches are implemented using Mach-Zehnder interferometers (MZI). Each pair of adjacent switches is connected by a long spiral waveguide and a shorter straight waveguide. The shorter waveguide serves as the reference arm. The delay difference between the long waveguide and the shorter waveguide is the delay for that stage. The first stage has a length difference Δ*L* between the long and shorter waveguides, corresponding to a delay of Δ*t* = Δ*L*/*v_g_*. The *N*-th stage sets the length difference between the long and shorter waveguides as 2(*N*-1)Δ*L*, corresponding to a delay of 2(*N*-1)Δ*t*. This design ensures to achieve delay changes from Δ*t* to 2(*N*-1)Δ*t* with a step size of Δ*t*. At the *N*+1 stage, thermo-optic refractive index modulation of the silicon waveguide is utilized to realize fine delay adjustment. By appropriately setting the length of the heated waveguide at the *N*+1 stage, *L_heated_*, a continuously tunable delay ranging from 0 to Δ*t* is achieved at this stage. In this design, combining the *N*+1 stage of thermally tuned silicon waveguides with the digital programmable delay tuning, the purpose of ultra-high precision continuous tunable delay is ultimately achieved. The length difference for step delay with optical switches and the length of the heated waveguides for continuously tunable delay are shown in Table S1.

The working principle of the thermo-optics fine delay adjustment is to change the refractive index of the waveguide by injecting current into the resistive heater along them, thereby changing the effective refractive index of optical mode. The relationship between the delay tunning and the temperature change is given as:

$$\Delta t=L_{heated}n_{g}\left(d_{n}/d_{T}\right)\Delta T/c$$

where *d_n_*/*d_T_* is the change in the refractive index of the TE_0_ mode versus the change in temperature. This coefficient is not only affected by the change in the refractive index of silicon, but also the change in the refractive index of the surrounding claddings. Δ*T* is the change in the temperature, and *L_heated_* is the length of fine-tuning waveguide region. In the experiment, a 1 ps delay corresponds to a voltage change of ~12.5 V as shown in Fig. S3. The voltage controlling accuracy of our home-made multi-channel voltage source is 1 mV. Thus, the designed delay controlling accuracy of the tunable TDL is about 0.08 fs. In future demonstration, the design of the thermo-optic fine-tunable delay line should be further optimized. Potential improvements include suspended silicon waveguides for thermal isolation, optimizing heating-electrode architecture, and using more efficient heating materials.

**S 5** **Potential** **scalability of the analog parallel processing**

The basic concept of the APP is to discretize analog input signal with optical frequency comb and then reassign the analog signal into 2*^N^* parallel sub-sequences through optical dynamic phase interfering. While an APP with two sub-channels is demonstrated in the main text, a larger scale implementation of APP with more sub-channels can also be realized. The architecture of the large-scale APP is illustrated in Fig. S4. The basic idea is to cascade DO-MZMs after each output of the previous DO-MZM for constituting more sub-channels. For each DO-MZM, the data rate of output sequences equals half of the input sequences. With cascaded configuration, the data rate of each sub-sequence output from the APP is 1/*NT_S_* after passing through *log*_2_*N* DO-MZMs. It means that the total data rate of the APP can be multiplied by scaling up. In addition, a TDL is added before each DO-MZM to maintain maximal and minimal intensity transmission levels for adjacent optical pulses. Each DO-MZM is driven by a driving signal and the frequency of driving signal is *f_S_*/2, *f_S_*/4… *f_S_*/2*^N^*, respectively.

A key point of large-scale APP is low-loss DO-MZMs since they are largely adopted in the architecture. With the maturity of the thin-film lithium niobate platform, the intensity modulator with a low loss of ≈0.5 dB is obtainable [2], guaranteeing the implementation of large-scale APP. Another factor determining the total data rate is the comb spacing of the optical frequency comb. The optical frequency comb with large comb spacing up to 180 GHz has been demonstrated [3], it indicates that the total data rate can be greatly increased.

As the number of sub-channels increases to 2*^N^*, the number of TDLs deployed before each DO-MZM also increases, as depicted in Fig. S4. The total number of TDLs goes to 2*^N^*-1, which would introduce cumulative loss. The insert loss of each TDL depends on its delay tuning range, the TDL with larger tuning range would result in greater insertion loss. When cascaded *log*_2_*N* DO-MZMs in each branch, the required delay tuning ranges of TDLs are *T_S_*, 2*T_S_*… 2*^N^*^-1^ *T_S_*, respectively. Since each TDL is composed of thermally tuned waveguides and cascading optical switches with step delays. The accumulated loss would increase with scale, which needs to be compensated by on-chip amplifier [4].

To expand total data rate and the number of sub-channels, the design of microwave driving signals becomes crucial. In this sense, the amplitude and phase of each microwave driver signal must be properly managed to optimize the parallelization performance. Besides, all microwave signals should be synchronized for coherence. Thus, a microwave-chip-based coherent multi-frequency microwave driver module is more conducive than microwave generators in future scaling. According to the requirements of microwave driver signals, the microwave driver module should include power splitters, low noise amplifiers, power amplifiers, band-pass filters, variable attenuators, and variable phase shifters to obtain sufficient gain and well-managed amplitude and phase. The power consumption of the multi-frequency microwave driver module is determined by the above chips. For reference, the power consumption of the microwave driver module in S.Ref. [5] is 15.8 W, which has one path 20 GHz, two path 10 GHz and four path 5 GHz driving signals. Besides, RF connections would also increase with the expanding of sub-channels. In future scalability, to minimize the constraint from size and routing of RF lines, multi-frequency microwave driver can be integrated in a single chip and packaged with the silicon photonic chip through advanced technologies such as flip-chip [6] or wafer-level package [7].

To match the parallel sub-sequences, the coefficients of multipliers should be decomposed in the same order and spatially divided into 2^N^ channels as well. As derived in Eq. (2) and Supplementary Note 1, each sub-sequence of signal vector and coefficient vector should interact with each other. Then the results of decomposed convolution or dot product are added in pairs to achieve the complete processing result. With the expanding of sub-channels, the sub-sequence of coefficient vector also increases. In other words, for the same original signal, requirements on speed and capacity of each parallel computing core are also reduced exponentially.

When expanding to 2^N^ sub-channels, the amplitude and phase uniformity become more difficult to maintain considering the mass production consistency. For amplitude adjustment, on-chip variable optical attenuators [8] can be added after outputs from DO-MZMs to compensate for the loss difference. Phase uniformity is also critical for our analog parallel processing, which can be optimized through on-chip TDLs.

In this part, we analyze the footprint of APP chip with large-scale expanding. As illustrated in Fig. S4, the architecture of the large-scale APP with 2^N^ sub-channels consists of an MZM, *log*_2_*N* DO-MZMs and *log*_2_*N* tunable TDLs. The required delay tuning ranges of TDLs are *T_S_*, 2*T_S_*… 2*^N^*^-1^ *T_S_*, respectively. According to the footprint of this demonstrated two-channel APP chip, the size of the MZM and single DO-MZM is 1.15×2.88 =3.31 mm^2^ and 1.24×2.85=3.534 mm^2^, respectively. The tunable TDL in the two-channel APP chip takes 0.645×2.82=1.819 mm^2^. When the number of sub-channels is set to 4, 8, and 16, the size is estimated to be 23.01 mm^2^, 66.25 mm^2^, and 210.94 mm^2^, compatible with current photonic integration technologies.

**S 6 Power consumption analysis**

The power consumption of the APP chip mainly comes from the thermal phase shifters of optical switches in the TDL and modulators. There are totally three optical switches in our chip, working either at the cross or the bar state. The total average power consumption of all the optical switches is ∼0.04 W. The power consumption of the MZM and the DO-MZM are ∼0.007 W and ∼0.36 W, respectively. Since the DO-MZM operates in the nonlinear region and the MZM operates in the small signal region, there is a difference in power consumption. The power consumption of the APP chip is ∼0.407 W, including the power from optical switches and modulators. In addition to on-chip components, the power consumption of optical frequency comb is ~20 W. With 0.01 W per photodetector and 0.24 W per ADC, we get the power consumption of 20.907 W for the entire system. which could be significantly reduced to less than 2 W when using cavity-less ultra-short optical pulses based on cascaded modulators to replace the optical frequency comb.

**S 7 Conceptual demonstration for detecting multiple targets**

The experimental setup is the same as Fig. 4a. We simulated the radar echo signal of detecting two targets and generated it through the AWG (Keysight Technologies, M8195A). The bandwidth of this echo signal is 6 GHz, from 2 GHz to 8 GHz. The echo signal is amplified by a power amplifier (CONNPHY CLN-0.1G20G-3040-S), and then fed into the APP chip. In the APP chip, the echo signal is temporally discretized and parallelized into two sub-sequences with lower data rate. The temporal waveforms output from two sub-channels are shown in Fig. S6a. The delay between adjacent data points in each sub-channel is 50 ps and the data rate for each sub-channel is 20 GHz. The reconstructed waveform is achieved through rearranging the data from two sub-channels one by one. The data rate of the reconstructed waveform is 40 GHz. The pulse compression is also conducted and the results are depicted in Fig. S6b. It is observed from zoom-in insets that the range difference between two targets is ~3 cm. Thus, the capability of the APP chip for detecting multiple targets at multiple distances is verified.

**Supplementary references**

1. S. Hong, L. Zhang, Y. Wang, et al, Ultralow-loss compact silicon photonic waveguide spirals and delay lines, Photon. Res. 10, 1-7 (2022).
2. N. Chen, K. Lou, Y. Yu, et al, High-Efficiency Electro-Optic Modulator on Thin-Film Lithium Niobate with High-Permittivity Cladding, Laser Photonics Rev. 17, 2200927 (2023).
3. H. Shu, L. Chang, Y. Tao, et al, Microcomb-driven silicon photonic systems, Nature, 605, 457–463 (2022).
4. Y. Liu, Z. Qiu, X. Ji, et al, A photonic integrated circuit–based erbium-doped amplifier, Science, 376, 1309-1313 (2022).
5. J. Li, N. Qian, S. Hua, et al, Optimization of optical signal-to-distortion ratio in a channel-interleaved photonic ADC via a coherent multi-frequency RF driver, Chin. Opt. Lett. 19, 083901 (2021).
6. P. Tumne, H. Wang, D. R. Shirley, et al, 2.5D silicon photonics interposer flip chip attach, IEEE 73rd Electronic Components and Technology Conference (ECTC), 1896-1902 (2023).
7. Y. Lin, S. Wu, W. Shen et al, An RDL-first fan-out wafer level package for heterogeneous integration applications, IEEE 68th Electronic Components and Technology Conference (ECTC), 349-354 (2018).
8. Z. Chen, L. Lu, W. Shan, et al, Silicon integrated microwave photonic beamformer, Optica, 7(9): 1162-1170, (2020).


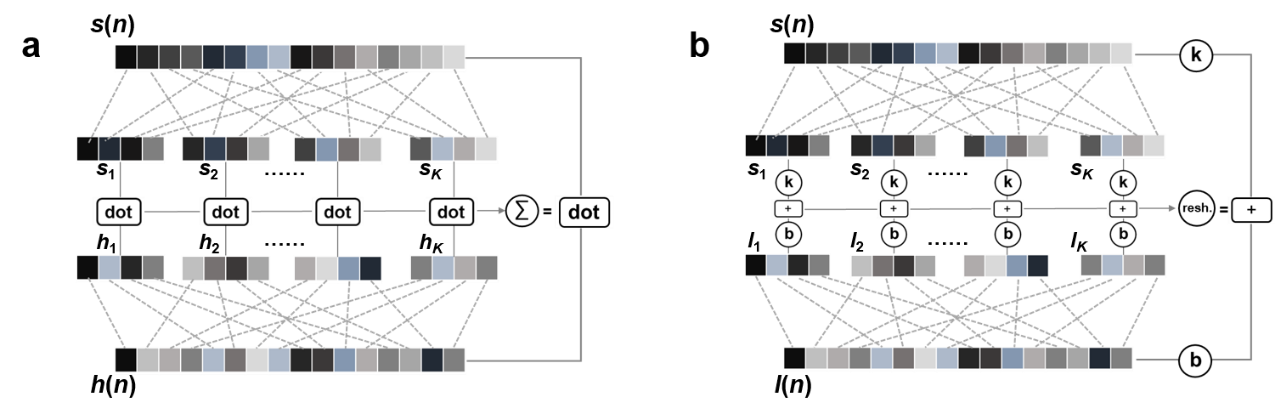


**Fig. S1: Schematic of calculation decomposing for analog parallel processing. a,** Decomposing of the dot product operation**. b,** Decomposing of the basic vector operation.


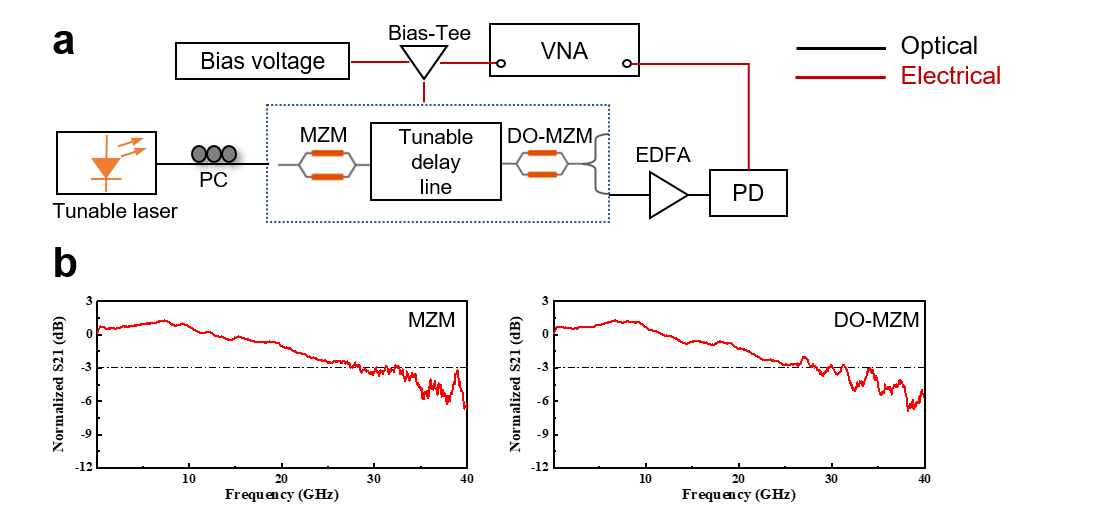


**Fig. S2: Characterization of the silicon EO modulator.** a, Experimental setup; b, Measured bandwidth of the on-chip silicon EO modulator. PC, polarization controller; EDFA, erbium-doped fiber amplifier; PD, photodetector; VNA: vector network analyzer.


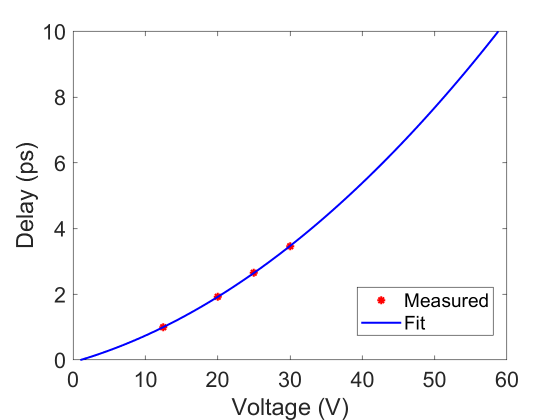
**Fig. S3: Characterization of the thermo-optics fine tunable delay line.**


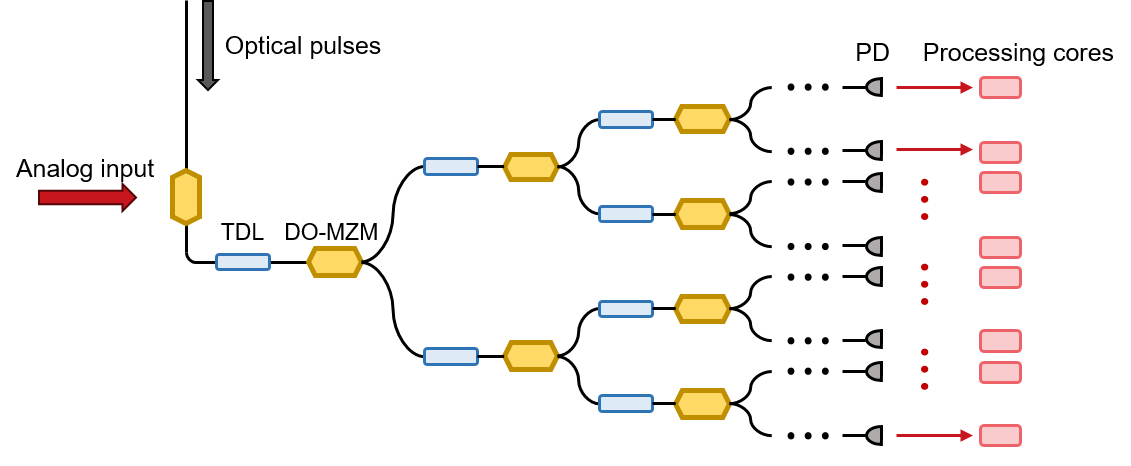


**Fig. S4: The architecture of the large-scale APP.** Cascading DO-MZMs after each output of the previous DO-MZM for constituting more sub-channels, the data rate of each sub-sequence output from the APP is 1/*NT_S_* after passing through *log*_2_*N* DO-MZMs.

**
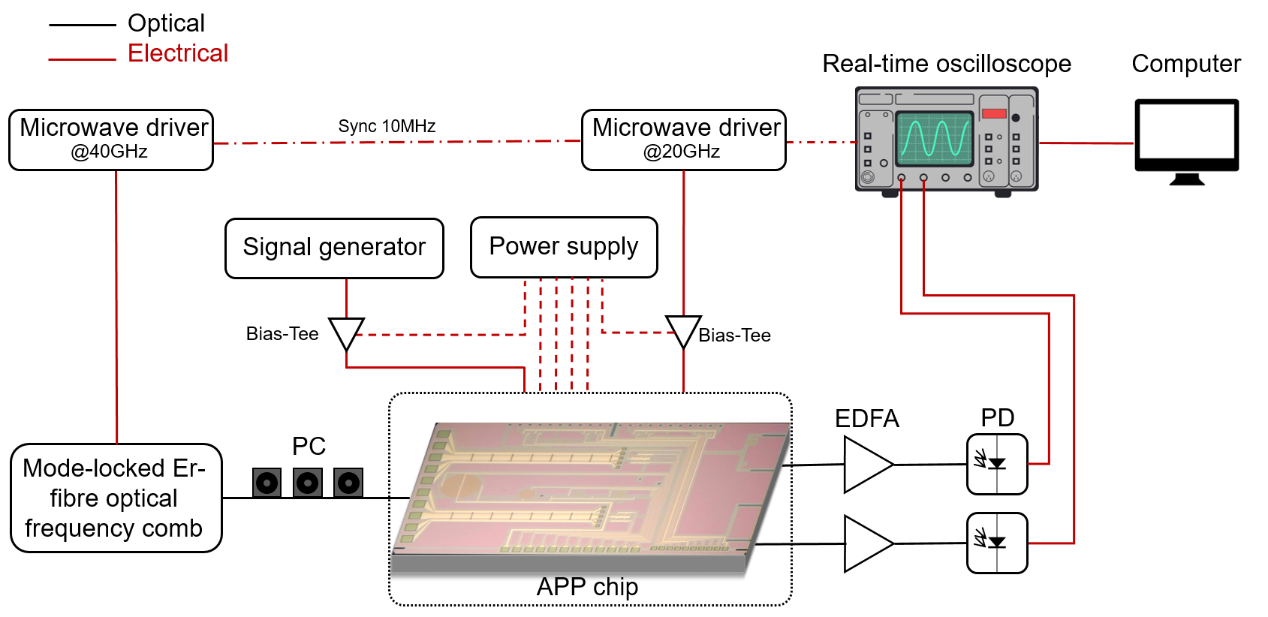
**

**Fig. S5: Experimental setup of analog parallel processing.** PC, polarization controller; EDFA, erbium-doped fiber amplifier; PD, photodetector; Sync: synchronization.

**
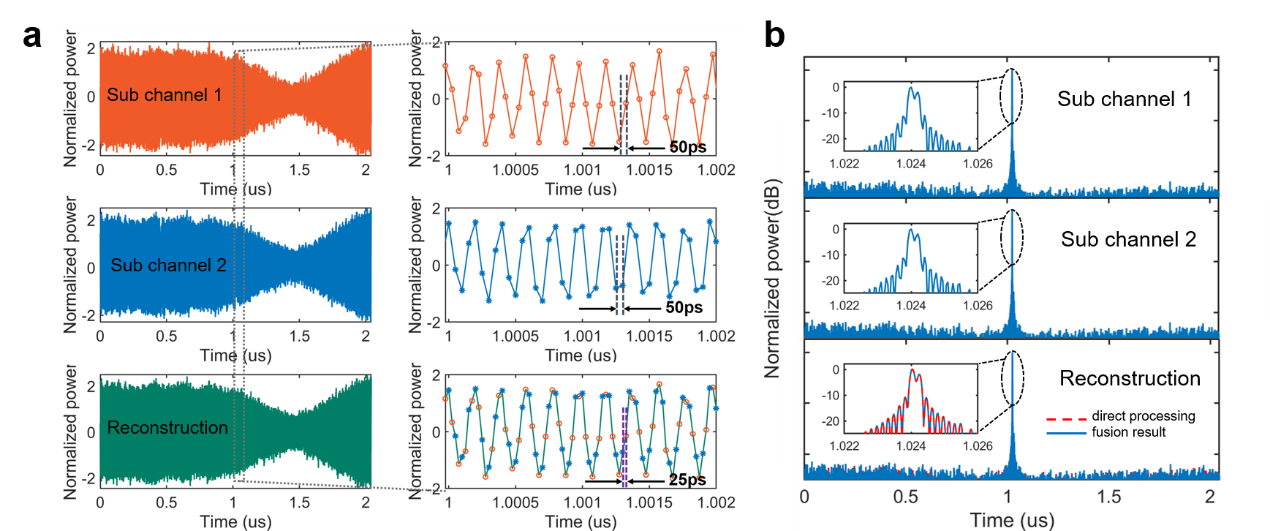
**

**Fig. S6: Experimental results of multiple targets detecting.** (a) The temporal waveforms from each sub-channel and reconstruction result. (b) Pulse compression results of each sub-channel; fusion result of two sub-channels (blue solid line in bottom); direct processing result (red dashed line in bottom).

**Table S1: The length difference for step delay with optical switches and the length of the heated waveguides for continuously tunable delay.**

| Stage | Type | Delay (ps) | Length (μm) |
| --- | --- | --- | --- |
| Stage1 | step delay | 10 | 793.5 |
| Stage2 | step delay | 20 | 1587.05 |
| Stage3 | continuously tunable delay | 1-10 | 43133.2 |
| Stage4 | continuously tunable delay | 0.1-1 | 4313.3 |
